# Evaluation of the Color Stability of 3D-Printed Crown and Bridge Materials against Various Sources of Discoloration: An In Vitro Study

**DOI:** 10.3390/ma13235359

**Published:** 2020-11-26

**Authors:** Ji-Won Shin, Jong-Eun Kim, Young-Jin Choi, Seung-Ho Shin, Na-Eun Nam, June-Sung Shim, Keun-Woo Lee

**Affiliations:** 1 Undergraduate Course, Yonsei University College of Dentistry, Yonsei-ro 50-1, Seodaemun-gu, Seoul 03722, Korea; caitlynlove@naver.com (J.-W.S.); cjh9217@naver.com (Y.-J.C.); 2 Department of Prosthodontics, Yonsei University College of Dentistry, Yonsei-ro 50-1, Seodaemun-gu, Seoul 03722, Korea; gomyou@yuhs.ac (J.-E.K.); shin506@prostholabs.com (S.-H.S.); jennynam5703@prostholabs.com (N.-E.N.); jfshim@yuhs.ac (J.-S.S.); 3 Department of Prosthodontics, Veterans Health Service Medical Center, 53 Jinhwangdo-ro 61-gil, Gangdong-gu, Seoul 05368, Korea

**Keywords:** 3-D printing, CAD-CAM, dental prosthesis, staining

## Abstract

Recent advances in three-dimensional (3D) printing have introduced new materials that can be utilized for dental restorations. Nonetheless, there are limited studies on the color stability of restorations using 3D-printed crowns and bridge resins. Herein, the color stability of conventional computer-aided design/computer-aided manufacturing (CAD/CAM) blocks and 3D-printing resins was evaluated and assessed for their degrees of discoloration based on material type, colorant types (grape juice, coffee, curry, and distilled water (control group)), and storage duration (2, 7, and 30 days) in the colorants. Water sorption, solubility, and scanning electron microscope (SEM) analyses were conducted. A three-way ANOVA analysis showed that all three factors significantly affected the color change of the materials. Notably, the discoloration (ΔE_00_) was significantly higher in all 3D printing resins (4.74–22.85 over the 30 days) than in CAD/CAM blocks (0.64–4.12 over the 30 days) following immersion in all colorants. 3D-printing resins showed color differences above the clinical limit (2.25) following storage for 7 days or longer in all experimental groups. Curry was the most prominent colorant, and discoloration increased in almost all groups as the storage duration increased. This study suggests that discoloration must be considered when using 3D printing resins for restorations.

## 1. Introduction

Recently, manufacturing restorations using computer-aided design/computer-aided manufacturing (CAD/CAM) has become an important process in dentistry and has replaced traditional methods in many areas. The CAD/CAM system, comprising optical scanners, the CAD software, and the manufacturing equipment, is gradually being used in dental clinics due to its advancement in technology [1,2]. Compared to the conventional dental restoration manufacturing process, this new method has simpler and more accurate procedures and better processing precision [1,2]. In addition, dentists and dental technicians can observe and communicate the design of the prosthesis digitally, and the data of the design can be stored as a digital file [3,4,5,6].

With the development of the CAD/CAM system, three-dimensional (3D) printing or additive manufacturing is rapidly becoming popular as well [7]. 3D printing technology is emerging as a new technology that overcomes the limitations of manufacture systems in dentistry, with the development of 3D printing materials and improvement of 3D printers [8,9]. 3D printing is used to manufacture biomaterials for dental treatment, surgery, and medical devices. In dentistry, it is mainly used to manufacture dental implants, orthodontic models, metal restorations, implant surgery guides, and temporary crowns [8,10,11]. The strengths of currently available tooth-shade 3D printing resins are lower than those of zirconia and lithium disilicate. Thus, they are mainly used for temporary or long-term provisional restorations [12,13].

In dental prosthesis, temporary restorations are important for successful treatments and are used for diagnostic purposes, stabilization of the occlusion and periodontium, aesthetics, oral hygiene, and pulp protection [14,15]. Polymer-based resins, including acrylic resins, bis-acryl composite resins, and composite resins are mainly used for temporary restorations [15,16]. Acrylic resins are inexpensive and easy to process; however, heat is generated during polymerization, and micro-leakages can occur due to polymerization shrinkage and the difference in its thermal expansion coefficient with enamel [17,18,19]. Bis-acryl resins have better mechanical properties than acrylic resin. They have a similar coefficient of thermal expansion with enamel, are more aesthetically pleasing and exhibit less polymerization shrinkage; however, they have low resistance to deformation and are expensive [18,20]. Recently, prefabricated polymethyl methacrylate (PMMA), used in milling devices, which are pre-polymerized in a well-controlled environment, showed good physical properties such as strength and material density. However, since the prosthesis is made in a subtractive manner, the consumption of material and milling burr are significant [21]. 3D-printing resins can be used by polymerizing a photopolymer material only on the part of the prosthesis to be produced; it has the advantages of low material consumption and economical use [13]. Consequently, interest in the workflow of manufacturing prostheses using additive manufacturing has increased recently.

In an aesthetic point of view, color matching between restorations and natural teeth is critical [12]. Even temporary restorations used in the dental restoration process require a quality comparable to that of natural teeth. Moreover, temporary restorations must be resistant to discoloration by external coloring factors such as food [22,23]. In particular, color stability should be maintained in clinical situations wherein temporary resin restorations are delivered for a long time, such as in orthodontic patients who attach the bracket to the resin prosthesis; in patients who wear the resin prosthesis for vertical dimension alteration, maintaining color stability for a long period of time is very important in maintaining the quality of life of patients [24,25].

Although manufacturing dental prostheses using 3D printing has become popular in recent years, studies that may serve as guidelines for intraoral applications of 3D printing resins are lacking. In particular, existing studies on the color stability of 3D printing resins exposed to various colorants is insufficient. Thus, this study aimed to evaluate the discoloration resistance and color stability of CAD/CAM block and 3D printing materials by evaluating color changes upon exposure to staining foods. The null hypothesis is that there is no difference in discoloration characteristics due to the restoration material used, discoloration based on the type of colorants used, or storage time of the materials in colorants.

## 2. Materials and Methods

In this study, three kinds of CAD/CAM blocks, namely, polycarbonate (Polycarbonate block, Line dental lab, Seoul, Korea), PMMA (Vipi block, Vipi, São Paulo, Brazil), and dispersed-filler composite (MAZIC Duro, Vericom Co., Chuncheon, Korea), and two kinds of 3D printing resins, i.e., Nextdent C&B (Vertex-Dental B.V., Soesterberg, The Netherlands), and denture teeth A2 resins (Formlabs Inc., Sommerville, MA, USA), were used (Table 1).

**Figure 1:**
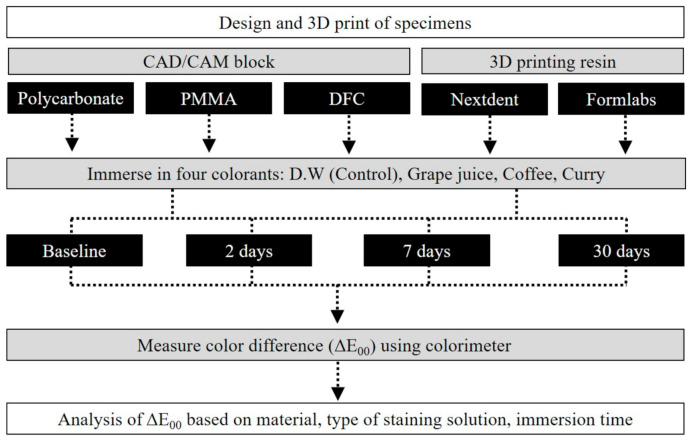
Overall experimental workflow of this study. PMMA—polymethyl methacrylate; DFC—dispersed-filled composite; DW—distilled water.

All procedures were conducted in the laboratory of Yonsei University College of Dentistry, Seoul, Korea, and the overall workflow of this study is illustrated in Figure 1.

To evaluate the color tones of the materials before and after storage in colorants, disk-shaped specimens, with a diameter of 10 mm and a thickness of 3 mm, were designed using a 3D modeling software (Rhino 5, Robert McNeel & Associates, Seattle, WA, USA) and exported as a file in Standard Tessellation Language (STL) format (Figure S1). CAD/CAM blocks, with a diameter of 98.5 mm and thickness of 18 mm, were cut and trimmed using a precision cutting machine (ASM100A, Okamoto Co., Tokyo, Japan) and diamond wheels (#400), respectively. For the 3D printing of the two types of 3D printing resins, the designed STL format file was imported into a slicing software (PreForm, Formlabs Inc., Sommerville, MA, USA), where a support structure was formed and 3D printing parameters were established. The thickness of each printing layer was set to 100 μm, and the support structure was attached to the bottom of the disk specimens. The two types of 3D printing resins were printed using Digital Light Processing (DLP) 3D printer with a 405-nm ultraviolet (UV) light emitting diode (LED) light (Nextdent ND5100, Vertex-Dental B.V., Soesterberg, The Netherlands) and Stereolithography (SLA) 3D printer with a 405-nm UV LED light and 250-mW laser power (Form3, Formlabs Inc., Sommerville, MA, USA). For the printing of Nextdent C&B resin and Denture Teeth A2 resin, built-in programmed parameters for each material in a compatible 3D printer were used. The printed specimens were washed using a washing machine (Twin Tornade, Medifive Co., Seoul, Korea) and 90% isopropyl alcohol. The post-curing process was conducted in accordance with the manufacturer’s recommended conditions in the UV post-curing equipment (CureM D102, Sona Global, Seoul, Korea). Then, support structures were removed from the printed specimens, and the remaining irregular structures on the surface were removed. A total of 200 specimens consisting of 40 specimens for each of the 5 types of materials were produced (Figure S2). The produced specimens were polished on both sides using carbon papers of up to 1200 grit under water cooling. The polished specimens were then cleaned for 30 s in an ultrasonic cleaner, and the cleaned specimens were stored in distilled water at 37 °C for 24 h.

Manufactured specimens of each material were randomly divided into four groups of 10 specimens for each colorant to analyze the effects of exposure to colorants on CAD/CAM blocks and 3D printing resins over time. For assignment of specimens used in each group, a random number between 0 and 1 was generated using the RAND function of Microsoft Excel 2016 (Microsoft Corporation, Redmond, WA, USA), and the specimens were randomized based on the number value obtained. The colorants used in this study were as follows: Grape juice (Grape 100, Del Monte Food Inc., Walnut Creek, CA, USA), coffee (Maxim mocha gold coffee, Dongsuh Food, Seoul, Korea), curry (Ottogi Curry hot, Ottogi Co., Anyang, Korea), and distilled water (Control group). Coffee solution was prepared by dissolving 11.7 g of coffee powder in 200 mL of warm water; the curry was prepared by dissolving 20 g curry in 200 mL of water. Distilled water and grape juice were used as they were delivered. All specimens were immersed in the prepared colorants and stored for 30 days inside a 37 °C incubator in a dark environment. A colorimeter (Minolta Cr321 Chromameter, Minolta, Osaka, Japan) was used to measure the color quantitatively; baseline color measurements were performed before specimen storage in the colorants. Color changes were measured at 2, 7, and 30 days after storage in the colorants. It is known that a 24-h in vitro incubation in the colorants simulates conditions similar to exposure to the colorants during food intake over ~30 days [26,27]. The maximum storage period of 30 days evaluated in this study is equivalent to approximately 2.5 years.

Color measurements were performed three times for each specimen, and the average value was recorded. L* is the lightness, whereas “a*” (green~magenta) and “b*” (blue~yellow) are the chromatic axes. L*, a*, and b* values measured at each time point were applied to the CIEDE2000 formula (ΔE_00_) to evaluate the changes in color tones caused by the colorants. The ΔE_00_ values were calculated using Equation (1):

(1) $$∆E_{00}=\;\sqrt{{\left(\frac{∆L}{K_{L}S_{L}}\right)}^{2}+{\left(\frac{∆C}{K_{C}S_{C}}\right)}^{2}+{\left(\frac{∆H}{K_{H}S_{H}}\right)}^{2}+R_{T}\left(\frac{∆C}{K_{C}S_{C}}\right)\left(\frac{∆H}{K_{H}S_{H}}\right)}$$

wherein $S_{L},\;S_{C}\;\mathrm{and}\;S_{H}$ are the functions to calibrate the absence of visual uniformity of CIELab formula on the direction of lightness (L), chroma (C) and hue (H). $K_{L},\;K_{C},\;\mathrm{and}\;K_{H}$ are the correction parameters of environment. L*, a*, and b* values were measured on a white background, and parametric values of *K_L_*, *K_C_*, and *K_H_* were set to 1. If the ΔE_00_ value is higher than 1.30, it is clinically perceptible, and if it does not exceed 2.25, it is assumed to be clinically acceptable [28,29].

Water sorption and solubility tests were also conducted. Disk-shaped specimens with a diameter of 15 mm and a thickness of 2 mm were designed (Figure S3) and produced (n = 5, each material), and the manufacturing of the specimens, using CAD/CAM block and the printing method of the 3D printed specimen, were the same as those for the discoloration test. Following specimens’ preparation, silica gel was placed in a desiccator where the drying process was initiated at 37 °C and continued until a constant weight was maintained during repeated weighing. The weight was measured with an accuracy of approximately 0.1 mg, and the weight of each group was measured when no further changes in weight ($m_{1}$) was observed. Following drying completion, the specimens were immersed in distilled water at 37 °C for 7 days after which the visible moisture on the surface was removed with air for 15 s, and weight was measured ($m_{2}$), 1 min later. The specimens were again subjected to reconditioning at 37 °C in a desiccator containing silica gel. Reconditioning proceeded until no change in weight was observed, in which the final value was recorded ($m_{3}$). The value of water sorption ($W_{\mathrm{SP}}$) and solubility ($W_{\mathrm{SL}}$) were calculated using Equations (2) and (3) as follows:

(2) $$W_{\mathrm{SP}}\left(\%\right)=\left(\frac{m_{2}-m_{1}}{m_{1}}\right)\times 100$$

(3) $$W_{\mathrm{SL}}\left(\%\right)=\left(\frac{m_{1}-m_{3}}{m_{1}}\right)\times 100_{\;}$$

For morphological analysis, three types of CAD/CAM blocks and two types of 3D printed resin specimens without surface polishing (having a diameter of 10 mm and a thickness of 2 mm) were sonically cleaned in distilled water prior to Pt coating for 60 s (Cressington sputter coater 208HR, Cressington Scientific Instruments, Watford, UK). The specimens were then examined using scanning electron microscopy (SEM; JEOL-7800F, JEOL, Tokyo, Japan) for qualitative (SEM images) analyses.

Statistical analysis was performed using IBM SPSS v25.0 software (IBM Corp., Armonk, NY, USA). The Levene’s test and the Shapiro–Wilk normality test evaluated homoscedasticity and test normality, respectively. A three-way ANOVA analyzed the effects of the CAD/CAM blocks and 3D printing materials used, source of discoloration, and storage period, on the changes in the material color tones. One-way ANOVA and Repeated measures ANOVA analyzed the effects of the materials used on color tone changes and application time. One-way ANOVA was used to evaluate water sorption and solubility for each material. Tukey’s test was performed for post-hoc analysis (α < 0.05).

## 3. Results

**Figure 2:**
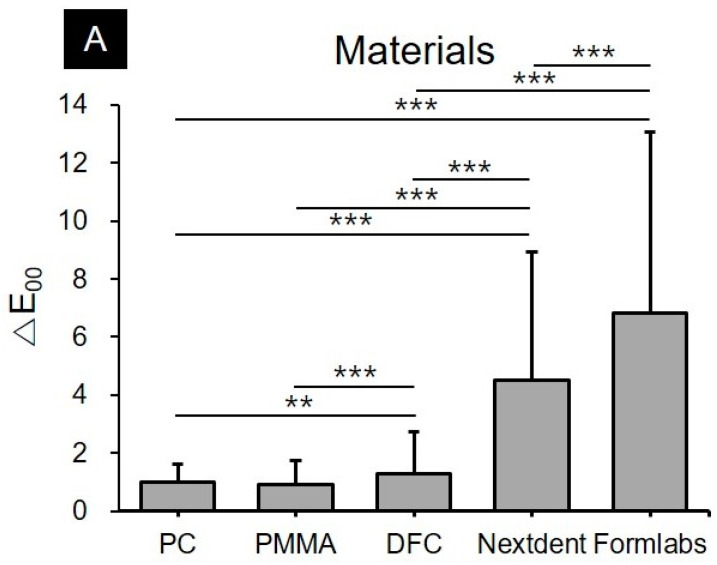

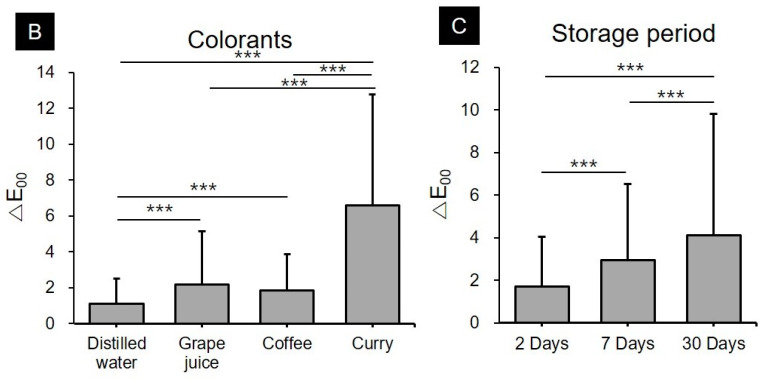
Results of the three-way ANOVA analysis of the changes in color tones according to the (**A**) materials used, (**B**) colorants, and (**C**) storage periods used in the study. The average was cumulative over the set of all other conditions. It is observed that color stability of 3D printing resins is low, and the changes in the color tone are significantly different depending on the type of colorant used and the storage period (mean + standard deviation). The double (**) asterisk represents a *p*-value ≤ 0.01 and the triple (***) asterisk represents a *p*-value ≤ 0.001.

Three-way ANOVA analysis showed that all three factors, including the type of material (F = 1595.5, *p* < 0.001), type of colorants (F = 1744.2, *p* < 0.001), and storage time in colorants (F = 540.1, *p* < 0.001) significantly influenced the discoloration of the materials. The interactions between the material used and the colorant (F = 343.6, *p* < 0.001), and the storage period (F = 186.2, *p* < 0.001), as well as the correlation between the colorants and the storage period (F = 37.0, *p* < 0.001), also significantly affected the discoloration of the materials. Interaction of the three factors (type of material, type of colorant, and storage time) also significantly affected discoloration (F = 43.7, *p* < 0.001) (Figure 2).

**Figure 3:**
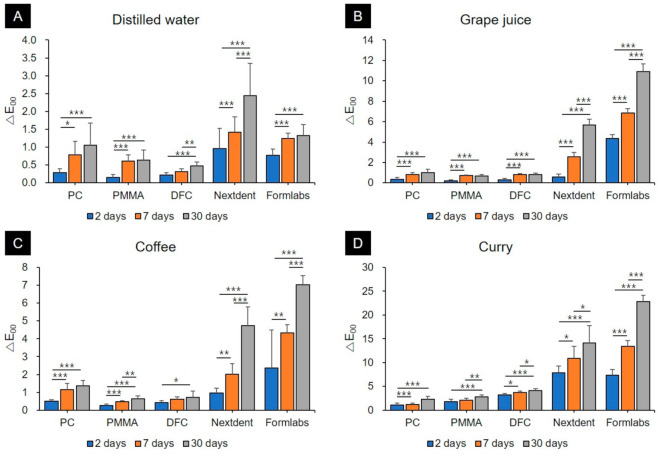
Graphs showing the differences in changes of color tones according to the type of colorant used (mean + standard deviation). (**A**) Distilled water, (**B**) Grape juice, (**C**) Coffee, (**D**) Curry. In each graph, the asterisk (*) represents a *p*-value ≤ 0.05, the double (**) asterisk represents a *p*-value ≤ 0.01 and the triple (***) asterisk represents a *p*-value ≤ 0.001.

Evaluation of the color stability of CAD/CAM blocks and 3D printing resins immersed in various colorants showed that both types of restoration materials and application time of the colorants had significant effects (*p* < 0.05). Significantly higher discolorations were observed in both 3D printing resins for all colorants, including distilled water, grape juice, coffee, and curry, than in the CAD/CAM blocks (Figure 3, Table S1).

Specimens stored in distilled water mostly showed ΔE_00_ values of 1.30 or less for the CAD/CAM block materials even after a storage period of 30 days. However, that of the Nextdent C&B resins exceeded 2.25 after a 30-days storage period.

CAD/CAM block materials including PC, PMMA, and DFC immersed in grape juice and coffee did not show ΔE_00_ values above 2.25 after 2, 7, and 30 days. Polycarbonate, when immersed in coffee for 30 days, showed a ΔE_00_ value of 1.36 ± 0.32, which was clinically perceptible, but not clinically acceptable. Among 3D printing resins, Nextdent C&B resins showed perceptible color changes with ΔE_00_ values of 2.58 ± 0.43 and 2.02 ± 0.59, respectively, after storage with grape juice and coffee, respectively, for 7 days. The ΔE_00_ values exceeded 1.30, showing perceptible color changes. Moreover, after a 30-days storage period, ΔE_00_ values of 5.68 ± 0.55 and 4.74 ± 1.05 were observed in grape juice and coffee, respectively, which exceeded the clinically acceptable range.

The degree of discoloration was much greater in all materials immersed in curry. In the PC and PMMA materials, ΔE_00_ values were less than 2.25 after 2 and 7 days of storage, which were clinically acceptable. However, their ΔE_00_ values were greater than 2.25 after a 30-days storage period. Discoloration was slightly more observed in DFC than in PC and PMMA. The ΔE_00_ value in DFC was 3.19 ± 0.35 after a 2-days storage period, which exceeded the clinically acceptable range. Moreover, the ΔE_00_ values in DFC were 3.68 ± 0.38 and 4.12 ± 0.45 after 7 and 30 days of storage, respectively. The 3D printing materials showed the most significant discoloration. Among them, the Nextdent C&B resin showed a ΔE_00_ value of 7.90 ± 1.37 after 2 days of storage. After a 30-days storage period, a ΔE_00_ value of 14.15 ± 3.61 was observed, which was the highest among the 30-days storage groups, indicating the greatest color change. Formlabs resins also had a ΔE_00_ value of 7.32 ± 1.26 after 2 days of storage and showed a maximum color change of 22.85 ± 1.24.

**Figure 4:**
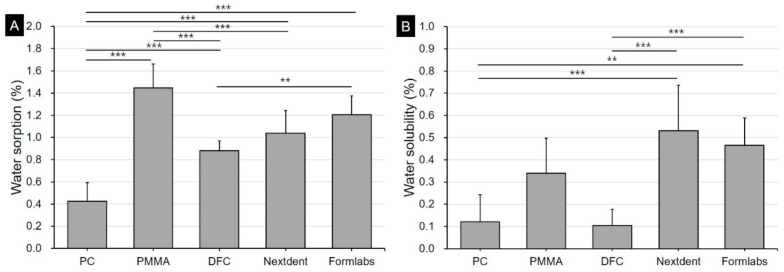
Graphs showing the differences in changes of water sorption and solubility according to the type of restorative materials (mean + standard deviation). (**A**) Water sorption, (**B**) Solubility. In each graph, the double (**) asterisk represents a *p*-value ≤ 0.01 and the triple (***) asterisk represents a *p*-value ≤ 0.001.

Water sorption and solubility results were illustrated in Figure 4. In the water sorption result, polycarbonate showed the lowest sorption at 0.43%. On the other hand, 3D printing resin showed a relatively high absorption of 1.04–1.21%, and prefabricated PMMA resin showed the highest water soprtion at 1.45%. In the solubility result, the two types of 3D printing resins showed the highest value of 0.47–0.53%, and the polycarbonate and DFC materials showed lower solubility of 0.12 and 0.07%, respectively.

**Figure 5:**
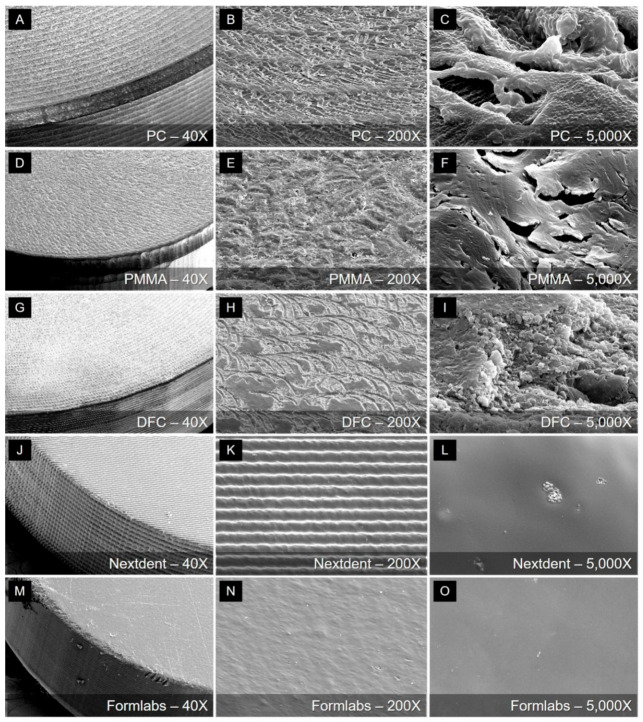
Qualitative scanning electron images of the surface topography of five restorative material specimens at (**A**,**D**,**G**,**J**,**M**) 40×, (**B**,**E**,**H**,**K**,**N**) 200×, and (**C**,**F**,**I**,**L**,**O**) 5000× magnifications. (**A**–**I**) Traces of bur passes were found on the surface of the specimen manufactured by the milling method. (**J**–**L**) Specimens of the Nextdent group made with a digital light processing 3D printer, and (**M**–**O**) Specimens of the Formlabs group made with a stereolithography 3D printer.

Qualitative (SEM images) analysis results are presented in Figure 5.

It is the surface of the unpolished specimen of five materials, and it can be seen that the shape of the surface is very different depending on the processing method. In the case of PC, PMMA, and DFC specimens manufactured through milling, it can be confirmed that traces of bur passes during the milling process remain (Figure 5B,E,H), and the microscopic surface was also very rough. It can be seen that the surface obtained through 3D printing is relatively smooth even in high magnification images (Figure 5L,O). A characteristic shape could be found on the surface of the 3D printing resin. In the case of Nextdent C&B resin printed by a DLP type printer, it can be confirmed that a characteristic pattern appears on the surface as it is printed using a micro mirror. The same surface features are also found on the side of the specimen. On the other hand, the Formlabs resin specimen printed by the SLA type 3D printer showed a relatively smooth surface.

## 4. Discussion

The recent development of CAD/CAM and 3D printing technology has led to the increased use of related materials, and the proportion of restorations made using materials with tooth shades is increasing. To obtain and maintain aesthetic results for short- and long-term periods, it is essential to evaluate the color consistency and stability of tooth-colored materials based on various environmental changes in the oral cavity. In this study, CAD/CAM blocks and 3D printing resins were immersed in food products with coloring factors (coffee, grape juice, and curry), in addition to distilled water (control group), to quantitatively assess the degree of discoloration according to the materials used and their storage periods in the colorants. As a result, it was observed that all 3D printing resins had lower color stability than all CAD/CAM block materials, and the discoloration in almost all experimental groups increased with storage time. Furthermore, among the sources of discoloration, curry caused the most discoloration in all materials. Therefore, the null hypothesis that there is no difference in discoloration characteristics depending on the restoration material and that there is no difference in discoloration according to the type of colorants used or storage time in the colorants were both rejected.

The degree of discoloration differed according to the material and storage time. First, there was a difference in the tendency of discoloration of CAD/CAM block materials depending on the type of colorant. However, it was clear that the discoloration of the 3D printing resins was greater than that of the CAD/CAM block materials in all groups. This suggests that the color stability of 3D printing resins is lower than that of the CAD/CAM resins. The clinically acceptable limit of color difference (ΔE_00_) is 2.25 [28,29]. In this study, a color difference above the acceptable limit was observed when 3D printing resins were immersed in grape juice for 7 days or more, and in coffee for 30 days or more, when PC and PMMA were immersed in curry for 30 days or more, and when DFC and 3D printing resins were immersed in curry for 2 days or more. In other words, 3D printing resins had a relatively noticeable color difference even after a short storage period in all colorants, and their color stability was significantly lower than that of the CAD/CAM block materials. In a recent study by Gruber et al. [30], color changes were evaluated for heat-polymerized resins, subtractive prefabricated PMMA resins, and 3D printing resins. There were no significant differences between the color stabilities of heat-polymerized resins and subtractive prefabricated PMMA resins; however, 3D printing resins showed strong color changes and very low color stability. This is in line with our study, confirming a greater extent of discoloration in 3D-printed resin compared to CAD/CAM resin blocks.

It is thought that there are several reasons for the low color stability of 3D printing resin specimens. Since 3D printing is based on the additive manufacturing (AM) method, layers exist in the surface microstructure [31,32,33]. Even on the SEM, the surface characteristics of the specimens were different based on the 3D printing method, such as SLA and DLP, but the microstructure could be observed as is reflected by the pattern structure on the surface. Since the DLP method utilizes the principle of 3D printing using a micro mirror, a slightly more characteristic pattern appeared on the surface, which may contribute to lowering color stability. However, as can be seen in Figure 5, the roughness of the unpolished surface is higher in the milling specimens than 3d printed specimens. Therefore, it is difficult to explain the low color stability of the 3D printed specimens due to the roughness exposed to the surface during manufacturing. Moreover, the low polymerization rate of 3D printing resins compared to other materials is another causative factor of low color stability [34]. PC, PMMA, and DFC materials are made by polymerizing in a high-temperature and high-pressure environment. Therefore, the polymerization rates in these materials are high, and their structures are compact [35]. In contrast, although 3D printing resins undergo post-curing processes after printing, their polymerization rates are relatively low [13]. A low polymerization rate may affect mechanical strength and biological processes as well as increase the possibility of discoloration due to poor surface integrity and affect surface deterioration due to the presence of residual monomers [36].

In addition, water sorption may also affect the discoloration characteristics. In our study, the water sorption of 3D printed resin tended to be higher than that of polycarbonate or dispersed-filled composite but lower than that of the pre-fabricated PMMA material. Berli et al. [37] had indicated that the water sorption of 3D printed resin was generally higher than that of pre-fabricated PMMA, which slightly differs from our findings. Berli et al. had also reported that the LuxaPrint Ortho Plus material had lesser water sorption than the pre-fabricated PMMA resin among the 3D printed resin materials used in their study. The authors indicated that even when using the same 3D printing method, each material showed different properties. Therefore, it appears that the water sorption rate of 3D printing resin can be influenced by other conditions such as material properties and other output parameters. Based on the results of this study, although water sorption alone cannot explain low color stability, it is considered a contributing factor [37,38].

In all experimental groups, changes in color tones gradually increased as the storage period increased. However, PC, PMMA, and DFC materials did not undergo clinically perceptible color changes after storage in distilled water, grape juice, and coffee; furthermore, the color change was slightly greater after storage in curry. In PC and PMMA, significant differences were observed only after 30 days of storage in curry. Nonetheless, a ΔE_00_ value > 2.25 was observed since the second day of storage in DFC. In contrast, the ΔE_00_ value exceeded 7 on the second day of storage in 3D printing resins, including Nextdent and Formlabs, with ΔE_00_ values reaching up to 20 after 30 days. In clinical situations where 3D printing resin restorations are used long-term, low color stability can cause aesthetic discomfort to patients. Thus, clinicians must be aware of the low color stability of 3D printing resins and be cautious in prescribing them to patients.

Barutcugil et al. [3], Aydin et al. [12], and Özarslan et al. [39] observed changes in the color differences and transparency by immersing DFC resin in beverage colorants such as distilled water, wine, coffee, and coke. These studies reported that the color difference in CAD/CAM resin was greater than the clinical limit after storage in wine and coffee for a long time. Poggio et al. [40,41] and E Silva et al. [42] quantitatively evaluated color stability by immersing composite resins and hybrid composite resins in colorants including saline, red wine, and coffee for 28 d. They observed that coffee caused the greatest discoloration in all aesthetic restorative materials, and that all color changes went beyond the clinical limit, regardless of the composition of each material, after a long storage period. In this study, the color changes in the CAD/CAM resin materials did not exceed the clinical limit except when they were immersed in curry, which is slightly different from the findings of other studies. Erdemir et al. [43] and Al-Dharrab et al. [44] assessed the color stability of nanofilled composite resins and micro hybrid composite resins after immersing them in energy drinks or sports drinks. It was observed that the colorants had different effects on the color stability of the material depending on the type of colorant used, storage time, and composition of the composite resin. These findings are consistent with the results of this study, where the colorants and storage time induced significant differences in color tones.

In this study, the color stability of the 3D printing resins was lower than that of the CAD/CAM resins. Color stability also differed between the two types of 3D printing resins used in this study. The differences in the composition of the resins are theorized to be the reason. However, most companies that produce 3D printing resins have patents to protect detailed ingredient combinations. Because it is difficult for general researchers to access such detailed information, a detailed analysis of these elements was not performed in this study. If detailed information is provided in the future, detailed analysis of the color stability of 3D printing resins may be feasible. Furthermore, the 3D printing resins used in this study were manufactured using dedicated 3D printers compatible with the resins. Setting parameters for 3D printing could also affect the surface quality during specimen fabrication. Such variables were not controlled in this study.

3D printing has become an important process in clinical dental practice. Application of 3D printing materials in dental industry will be more active in the future. Thus, additional studies on ways to improve printing methods, post-curing processes, and the materials themselves, are essential. In addition to the two types of 3D printing resins used in this study, which are the Nextdent C&B and the Formlabs denture teeth resins, various types of other 3D printing resins must also be evaluated for color stability. This will allow more detailed analysis of the effect of different resin ingredients and compositions on color stability. Moreover, as mentioned earlier, one of the causes of discoloration is the low polymerization rate of 3D printing materials. The rate may vary depending on the post-curing duration and methodology. Therefore, a follow-up study on the color stability of 3D printing resins according to different parameters of 3D printing and post-curing treatments such as the light source or temperature used would be necessary. In-depth research in these areas will increase the reliability and predictability in the dental treatment process using 3D printed crown and bridge materials.

## 5. Conclusions

1. The color stability of 3D printing resins such as the Nextdent C&B and Formlabs teeth resins was much lower than that of CAD/CAM block materials, which includes polycarbonate, polymethyl methacrylate, and dispersed-filled composite.
2. Various colorants significantly discolored the materials compared to distilled water. Among them, curry caused the most discoloration.
3. A greater discoloration was observed as the storage period increased.

## Figures and Tables

**Table 1:** Computer-aided design/computer-aided manufacturing (CAD/CAM) blocks and 3D printing resins used in the study.

Product	Component	Manufacturer
Polycarbonate block ^‡^	Polycarbonate, nanosilica filler, glass fiber, alkoxysilane	Line dental lab, Seoul, Korea
Vipi block monocolor	High cross-linked Polymethyl methacrylate (PMMA) resin	Dental VIPI Ltd., Sao Paulo, Brazil
MAZIC Duro	Composite resin material (BisGMA, TEGDMA)with 77 wt % silica, zirconia, and barium glass nanoparticles	Vericom, Anyang, Korea
Nextdent C&B	Methacrylic oligomers, Phosphine oxides	Nextdent, Soesterburg, The Netherlands
Denture Teeth A2 resin	Methacrylate monomer, Diurethane dimethacrylate, Propylidynetrimethyl trimethacrylate	Formlabs Inc., Somerville, MA, USA

^‡^ The polycarbonate CAD/CAM disk used in this study is a prototype and has yet to be marketed.

## Supplementary Materials

The following are available online at https://www.mdpi.com/1996-1944/13/23/5359/s1, Figure S1: Design image of specimen used for discoloration test, Figure S2: Photo image after manufacturing of the specimen used in this study, Figure S3: Design image of specimen used for water sorption and solubility test, Table S1: Average ΔE_00_ values of the CAD/CAM materials and 3D printing resins.
