# Supplementary material for: Evaluation of the Color Stability of 3D-Printed Crown and Bridge Materials against Various Sources of Discoloration: An In Vitro Study
Source: Materials (Basel). 2020 Nov 26;13(23):5359. doi: 10.3390/ma13235359 (PMC7731151; doi:10.3390/ma13235359)
Supplement: Supplementary file 1 [file materials-13-05359-s001.pdf]

materials

MDPI

Supplementary Materials

Evaluation of the Color Stability of 3D-Printed Crown and Bridge Materials Against Various Sources of Discoloration: An In Vitro Study

Ji-Won Shin [1,†], Jong-Eun Kim [2,†], Young-Jin Choi [1], Seung-Ho Shin [2], Na-Eun Nam [2], June-Sung Shim [2] and Keun-Woo Lee [2,3,*]

[1] Undergraduate Course, Yonsei University College of Dentistry, Yonsei-ro 50-1, Seodaemun-gu, Seoul 03722, Korea; caitlynlove@naver.com (J.-W.S.); cjh9217@naver.com (Y.-J.C.)
[2] Department of Prosthodontics, Yonsei University College of Dentistry, Yonsei-ro 50-1, Seodaemun-gu, Seoul 03722, Korea; gomyou@yuhs.ac (J.-E.K.); shin506@prostholabs.com (S.-H.S.); jennynam5703@prostholabs.com (N.-E.N.); jfshim@yuhs.ac (J.-S.S.)
[3] Department of Prosthodontics, Veterans Health Service Medical Center, 53 Jinhwangdo-ro 61-gil, Gangdong-gu, Seoul 05368, Korea
* Correspondence: kwlee@yuhs.ac; Tel.: +82-2-2228-3157
† These authors contributed equally to this work as first authors.

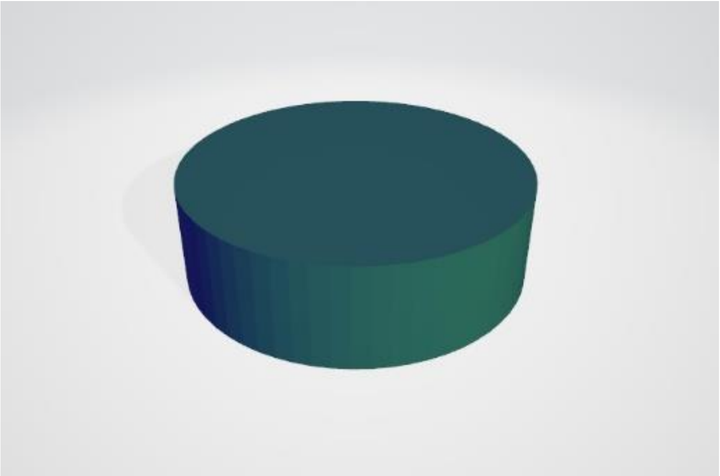

Figure S1. Disk-shaped specimens, with a diameter of 10 mm and a thickness of 3 mm, were designed using a 3D modeling software for discoloration test.

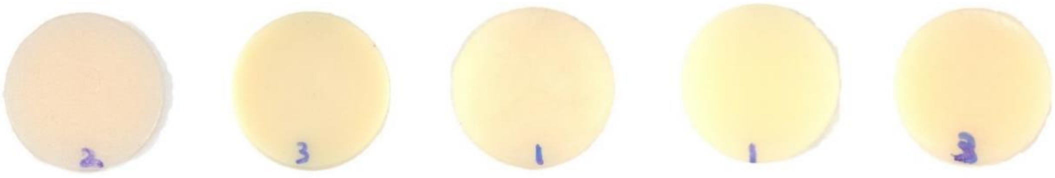

Figure S2. Five kinds of specimens of restoration materials used in this study. From left: PC, PMMA, DFC, Nextdent, Formlabs.

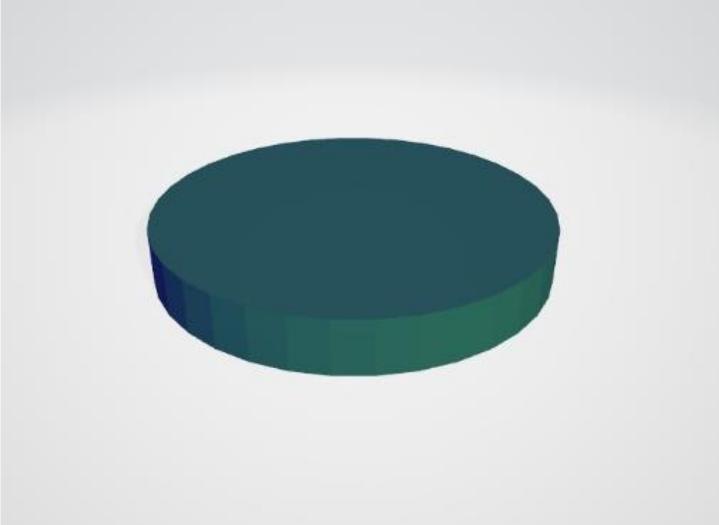

Figure S3. Disk-shaped specimens, with a diameter of 15 mm and a thickness of 2 mm, were designed using a 3D modeling software for water sorption and solubility test.

Table S1. Average $\triangle E_{00}$ values of the CAD/CAM materials and 3D printing resins after 2, 7, and 30 days of storage in the respective colorants.

Colorants	Days	PC (E_{00} ± SD)	PMMA (E_{00} ± SD)	DFC (E_{00} ± SD)	Nextdent (E_{00} ± SD)	Formlabs (E_{00} ± SD)
DW	2	0.29 ± 0.11 ^{A,a}	0.15 ± 0.08 ^{A,a}	0.22 ± 0.07 ^{A,a}	0.96 ± 0.57 ^{B,a}	0.77 ± 0.18 ^{B,a}
	7	0.79 ± 0.36 ^{B,b}	0.61 ± 0.17 ^{A,b}	0.32 ± 0.08 ^{A,a}	1.42 ± 0.43 ^{B,b}	1.25 ± 0.15 ^{B,b}
	30	1.06 ± 0.61 ^{B,b}	0.64 ± 0.29 ^{AB,b}	0.47 ± 0.11 ^{A,b}	2.44 ± 0.91 ^{C,c}	1.33 ± 0.30 ^{B,b}
Grape juice	2	0.34 ± 0.18 ^{A,a}	0.20 ± 0.11 ^{A,a}	0.28 ± 0.17 ^{A,a}	0.61 ± 0.26 ^{B,a}	4.35 ± 0.36 ^{C,a}
	7	0.84 ± 0.16 ^{A,b}	0.71 ± 0.07 ^{A,b}	0.84 ± 0.08 ^{A,b}	2.58 ± 0.43 ^{B,b}	6.86 ± 0.43 ^{C,b}
	30	0.99 ± 0.35 ^{A,b}	0.66 ± 0.17 ^{A,b}	0.80 ± 0.14 ^{A,b}	5.68 ± 0.55 ^{B,c}	10.93 ± 0.74 ^{C,c}
Coffee	2	0.51 ± 0.08 ^{B,a}	0.25 ± 0.08 ^{A,a}	0.43 ± 0.11 ^{B,a}	0.95 ± 0.27 ^{C,a}	2.37 ± 2.12 ^{D,a}
	7	1.15 ± 0.35 ^{B,b}	0.48 ± 0.05 ^{A,b}	0.60 ± 0.14 ^{A,ab}	2.02 ± 0.59 ^{C,b}	4.33 ± 0.45 ^{D,b}
	30	1.36 ± 0.32 ^{B,b}	0.64 ± 0.16 ^{A,c}	0.71 ± 0.36 ^{A,b}	4.74 ± 1.05 ^{C,c}	7.02 ± 0.51 ^{D,c}
Curry	2	1.13 ± 0.32 ^{A,a}	1.81 ± 0.52 ^{B,a}	3.19 ± 0.35 ^{C,a}	7.90 ± 1.37 ^{D,a}	7.32 ± 1.26 ^{D,a}
	7	1.22 ± 0.31 ^{A,a}	2.10 ± 0.39 ^{A,a}	3.68 ± 0.38 ^{A,b}	10.92 ± 2.45 ^{B,b}	13.47 ± 1.17 ^{B,b}
	30	2.28 ± 0.63 ^{A,b}	2.79 ± 0.38 ^{A,b}	4.12 ± 0.45 ^{B,c}	14.15 ± 3.61 ^{C,c}	22.85 ± 1.24 ^{D,c}

In each graph, different uppercase letters indicate the significant differences in the color change based on the materials used in the same row, and different lowercase letters indicate the significant differences in the color change according to the storage period in the same colorants ($P < 0.05$).
